# Simultaneous State and Parameter Estimation Methods Based on Kalman Filters and Luenberger Observers: A Tutorial & Review

**DOI:** 10.3390/s25227043

**Published:** 2025-11-18

**Authors:** Amal Chebbi, Matthew A. Franchek, Karolos Grigoriadis

**Affiliations:** Mechanical Engineering Department, Cullen College of Engineering, University of Houston, Houston, TX 77204, USA; mfranchek@uh.edu (M.A.F.); karolos@uh.edu (K.G.)

**Keywords:** simultaneous state and parameter estimation, Kalman Filters, Luenberger Observers, Extended Kalman Filter, Unscented Kalman Filter, Cubature Kalman Filter, Ensemble Kalman Filter, high-gain observers, sliding mode observers, adaptive observers, dynamic systems

## Abstract

Simultaneous state and parameter estimation is essential for control system design and dynamic modeling of physical systems. This capability provides critical real-time insight into system behavior, supports the discovery of underlying mechanisms, and facilitates adaptive control strategies. Surveyed in this review paper are two classes of state and parameter estimation methods: Kalman Filters and Luenberger Observers. The Kalman Filter framework, including its major variants such as the Extended Kalman Filter (EKF), Unscented Kalman Filter (UKF), Cubature Kalman Filter (CKF), and Ensemble Kalman Filter (EnKF), has been widely applied for joint and dual estimation in linear and nonlinear systems under uncertainty. In parallel, Luenberger observers, typically used in deterministic settings, offer alternative approaches through high-gain, sliding mode, and adaptive observer structures. This review focuses on the theoretical foundations, algorithmic developments, and application domains of these methods and provides a comparative analysis of their advantages, limitations, and practical relevance across diverse engineering scenarios.

## 1. Introduction

State estimation is fundamental to a broad range of engineering and scientific fields [1,2,3,4], as it involves inferring non-measurable internal states of a system. These states are essential for understanding, predicting, and controlling system performance [5,6,7], yet they often remain inaccessible due to sensor limitations, challenging environmental conditions [1], or the inherent system complexity. To address these challenges, various state estimation techniques have been developed, enabling the inference of internal states from available measurement data [8]. Among the widely used techniques are the Kalman Filter and its nonlinear extensions [9,10,11,12,13] and the Luenberger observer [14] for linear systems and its nonlinear system extensions [15,16,17,18].

Traditionally, state estimation techniques assume that model parameters are either (i) known a priori or (ii) unknown but constant throughout the estimation horizon (i.e., time-invariant) [15]. While this simplifies system modeling and analysis, it fails to account for the complexities of real-world systems where parameters may be uncertain or time-varying. In practice, model parameters often change over time due to environmental variations, operational shifts, or gradual system degradation. Neglecting these dynamics can lead to distorted representations of system behavior and inaccurate state predictions, as states and parameters are inherently interdependent. Therefore, it is crucial to employ methods capable of simultaneously estimating both system states and time-varying system parameters, enabling a more accurate and comprehensive characterization of system dynamics.

Extensive research has been devoted to state estimation in systems with known parameters, including numerous comparative analyses of classical filters and observers. Studies evaluating the Extended, Unscented, Cubature, and Ensemble Kalman Filters in nonlinear systems [19,20,21] have highlighted important trade-offs between estimation accuracy, numerical stability, and computational complexity, while comparative investigations of deterministic observers and adaptive Kalman filters under various noise and dynamic conditions [22,23] have provided valuable insights into estimator performance. However, these works primarily focus on state estimation alone and do not explicitly address the challenges associated with estimating system parameters that are uncertain or time-varying. Despite numerous review papers on Kalman filtering and observer design, to the best of the authors’ knowledge, no comprehensive review unifies and compares methods developed for simultaneous state and parameter estimation across both stochastic (Kalman-based) and deterministic (observer-based) frameworks. The present work therefore fills this gap by providing a structured, tutorial-style review that integrates theoretical foundations, algorithmic developments, and comparative perspectives on the principal approaches to concurrent state and parameter estimation.

The manuscript structure is as follows: Presented in Section 2 are Kalman filter-based state and parameter estimation methods for both linear and nonlinear systems, along with their modified versions for joint state-parameter estimation. It also includes a comparative analysis of these methods based on the reviewed literature and highlights their key limitations. Developed in Section 3 is a detailed examination of observer-based methods and their applications in state-parameter estimation. Provided in Section 4 a comparative discussion of Kalman-based and observer-based approaches. Finally, summarized in Section 5 are the key insights from the reviewed methods followed by the concluding remarks and future research directions.

## 2. State and Parameters Estimation: Kalman-Based Methods

The Kalman filter, introduced by Rudolf E. Kalman [24], is a widely used algorithm for estimating the internal states of linear dynamic systems from noisy or incomplete measurement data [25]. It operates recursively, processing a sequence of measurements over time to predict and refine state estimates. By accounting for uncertainties in both the system model and measurements, the Kalman filter produces optimal estimates of the system’s current state. Its strength lies in its ability to iteratively improve predictions by integrating new data, thereby reducing uncertainty over time. The filter operates in two key steps: the prediction (propagation) step, where the state estimate and covariance are projected forward in time, and the update (correction) step, where new measurements are incorporated to adjust the state estimate and minimize estimation error [26]. This recursive framework makes the Kalman filter highly effective for real-time applications in various fields, including control systems, navigation, and signal processing.

To describe the Kalman filter, consider the stochastic system

(1) $$x_{k+1}=F_{k}x_{k}+B_{k}u_{k}+w_{k},\;w_{k}∼N\left(0,Q_{k}\right),$$

(2) $$y_{k}=H_{k}x_{k}+v_{k},\;v_{k}∼N\left(0,R_{k}\right),$$

where $x_{k}$ is the state, $u_{k}$ the input, and $y_{k}$ the measurement. $F_{k},B_{k},H_{k}$ are the state transition, input, and observation matrices; $Q_{k}$ and $R_{k}$ are the process and measurement noise covariances. Note that the time-invariant case is recovered by setting $F_{k}=F$, $B_{k}=B$, $H_{k}=H$, $Q_{k}=Q$, and $R_{k}=R$.

The Kalman filter algorithm uses the state transition in (1) to predict the states at time step *k*, incorporating the process noise as zero-mean Gaussian noise with covariance Q. These predictions are used to compute the predicted system output through the measurement model in (2). The uncertainties are captured in the error covariance matrix P, which influences the calculation of the Kalman gain. The Kalman gain optimally balances the predicted states and measured data by weighting them according to the noise statistics represented in the Q and R matrices. This recursive process improves the system behavior estimates.

The Kalman filter process is summarized in Table 1, where ${\hat{x}}_{k}^{-}$ represents the predicted state estimate, ${\hat{x}}_{k}^{+}$ is the updated state estimate, $P_{k}^{-}$ is the predicted error covariance, $P_{k}^{+}$ is the updated error covariance, $u_{k-1}$ is the control input, $y_{k}$ is the measurement at time *k*, ${\tilde{y}}_{k}$ is the measurement residual, and $K_{k}$ is the Kalman gain. I is the identity matrix, employed in the update step to adjust the error covariance.

The use of the Kalman filter for simultaneous state estimation and parameter identification was first explored in the early 1970s [27,28,29,30]. Parameter identification involves utilizing the Kalman filter in a dynamically evolving system, where the state variables are augmented to incorporate the parameters being estimated [31]. This approach, known as joint state and parameter estimation, allows for the simultaneous estimation of both system states and parameters within a single filtering process, recursively updating the estimates over time as new measurements become available.

The dual state-parameter estimation is an alternative technique [32]. In this approach [33,34], the state vector x and the parameter vector θ are treated separately using two distinct concurrently executed filters: one for state estimation and the other for parameter estimation. This approach enables each filter to specialize its task, potentially improving accuracy and robustness. Other approaches combine a Kalman-based filter for state estimation with parameter estimation algorithms such as Least Squares Estimation (LSE) [35,36] or Recursive Least Squares Estimation (RLSE) [37] for parameter estimation. These hybrid solutions have the Kalman filter sequentially update the state estimates while the parameters are periodically updated using a batch or recursive least squares approach [15].

### 2.1. Kalman-Based Filters

This section presents the different variants of Kalman-based algorithms and their adaptations for both joint and dual state-parameter estimation. A comparative analysis is also provided, drawing on representative applications and published studies to evaluate the performance of these approaches in simultaneous state and parameter estimation tasks.

#### 2.1.1. Extended Kalman Filter

The Extended Kalman Filter (EKF) is a nonlinear extension of the standard Kalman Filter, designed to estimate the states of systems with nonlinear dynamics and measurements [38]. It accomplishes this by employing a Taylor series expansion to linearize the nonlinear system dynamics around the current state estimate, allowing it to effectively handle the complexities of nonlinear behavior.

To illustrate the EKF, consider the following discrete-time nonlinear state-space model, described by the state and measurement equations [1]

(3) $$\begin{array}{cc} x_{k} & =f\left(x_{k-1},u_{k-1}\right)+w_{k-1} \end{array}$$

(4) $$\begin{array}{cc} y_{k} & =h\left(x_{k}\right)+v_{k} \end{array}$$

where $x_{k}$ and $y_{k}$ are the state and the measurement vectors, respectively. The process noise $w_{k-1}$ and the measurement noise $v_{k}$ are assumed to be zero-mean with covariance matrices $Q_{k}$ and $R_{k}$, respectively. The functions *f* and *h* represent the nonlinear state transition function and the nonlinear measurement function, respectively. Defining the Jacobians $F_{k-1}$ and $H_{k}$ as

**State Transition Jacobian**

(5) $$F_{k-1}={\left.\frac{\partial f}{\partial x}\right|}_{{\hat{x}}_{k-1|k-1},u_{k-1}}$$

**Measurement Transition Jacobian**

(6) $$H_{k}={\left.\frac{\partial h}{\partial x}\right|}_{{\hat{x}}_{k|k-1}}$$

where $F_{k}$ and $H_{k}$ are the Jacobian matrices of the nonlinear state transition function *f* and measurement function *h*, evaluated at the estimated state ${\hat{x}}_{k|k}$ with control input $u_{k}$, and the predicted state ${\hat{x}}_{k|k-1}$, respectively.

The EKF is executed through the following two recursive steps [1], as described herein.

**State and Covariance Estimate Predictions:** In the prediction step, the state estimate ${\hat{x}}_{k|k-1}$ is projected forward in time using the nonlinear state transition function $f(x_{k-1},u_{k-1})$ based on the previous state estimate ${\hat{x}}_{k-1|k-1}$ and the current control input $u_{k}$. Simultaneously, the covariance estimate $P_{k|k-1}$ is predicted by propagating the previous covariance $P_{k-1|k-1}$ through the state transition matrix $F_{k}$, with the addition of the process noise covariance $Q_{k}$ to account for uncertainty

(7) $$\begin{array}{cc} {\hat{x}}_{k|k-1} & =f({\hat{x}}_{k-1|k-1},u_{k}) \end{array}$$

(8) $$\begin{array}{cc} P_{k|k-1} & =F_{k}P_{k-1|k-1}F_{k}^{⊤}+Q_{k} \end{array}$$

**State and Covariance Estimate Update:** In the update step, the Kalman gain $K_{k}$ is computed, which weighs the new measurement $y_{k}$ relative to the predicted measurement derived from the nonlinear measurement function $h(x_{k})$. This gain is used to adjust the predicted state estimate ${\hat{x}}_{k|k-1}$, resulting in the updated state estimate ${\hat{x}}_{k|k}$. The covariance estimate is also updated by incorporating the measurement, reducing uncertainty in the state estimate. The updated covariance $P_{k|k}$ reflects the reduced uncertainty after considering the new measurement

(9) $$\begin{array}{cc} K_{k} & =P_{k|k-1}H_{k}^{⊤}{\left(H_{k}P_{k|k-1}H_{k}^{⊤}+R_{k}\right)}^{-1} \end{array}$$

(10) $$\begin{array}{cc} {\hat{x}}_{k|k} & ={\hat{x}}_{k|k-1}+K_{k}\left(y_{k}-h\left({\hat{x}}_{k|k-1}\right)\right) \end{array}$$

(11) $$\begin{array}{cc} P_{k|k} & =\left(I-K_{k}H_{k}\right)P_{k|k-1} \end{array}$$

In Equations (7)–(11), $K_{k}$ represents the Kalman gain, which determines the weight given to the new measurement versus its estimate during the update process. The term ${\hat{x}}_{k|k-1}$ denotes the a priori state estimate, predicted before incorporating the current measurement, while ${\hat{x}}_{k|k}$ is the a posteriori state estimate, updated after accounting for the measurement. Similarly, $P_{k|k-1}$ is the a priori covariance matrix, representing the uncertainty in the state prediction before the update, and $P_{k|k}$ is the a posteriori prediction error covariance matrix, which reflects the reduced uncertainty after the measurement has been processed. This process allows the Kalman filter to refine both the state estimate and the uncertainty (covariance) using incoming measurements, iteratively improving the accuracy of the system’s understanding as new data becomes available.

#### 2.1.2. Unscented Kalman Filter

The Extended Kalman Filter approximates nonlinear systems by linearizing them around the current state estimate, whereas the Unscented Kalman Filter (UKF) directly processes the system’s nonlinear functions. By avoiding the approximation errors inherent in the EKF’s linearization, by instead propagating a set of sample points, called sigma points, through the system’s true nonlinear dynamics, the UKF offers greater accuracy, particularly in highly nonlinear and complex systems. This makes the UKF a more attractive choice in scenarios where the EKF’s performance is constrained. The UKF algorithm involves generating sigma points, predicting the state and covariance, incorporating new measurements, and refining the state estimate. The detailed steps are outlined as follows [39].

**Initialization**:

Begin by initializing the state estimate ${\hat{x}}_{0}$ and the covariance $P_{0}$

(12) $$\begin{array}{cc} {\hat{x}}_{0} & =E[x_{0}] \end{array}$$

(13) $$\begin{array}{cc} P_{0} & =E\left[\left(x_{0}-{\hat{x}}_{0}\right){\left(x_{0}-{\hat{x}}_{0}\right)}^{⊤}\right] \end{array}$$

**Sigma Points Generation:** Generate sigma points $\chi_{k-1}$ based on the current state estimate ${\hat{x}}_{k-1}$ and covariance $P_{k-1}$

(14) $$\begin{array}{cc} \chi_{k-1}^{\left(0\right)} & ={\hat{x}}_{k-1} \end{array}$$

(15) $$\begin{array}{cc} \chi_{k-1}^{\left(i\right)} & ={\hat{x}}_{k-1}+{\sqrt{\left(L+\lambda \right)P_{k-1}}}_{i},\;i=1,\ldots ,L \end{array}$$

(16) $$\begin{array}{cc} \chi_{k-1}^{\left(i\right)} & ={\hat{x}}_{k-1}-{\sqrt{\left(L+\lambda \right)P_{k-1}}}_{i-L},\;i=L+1,\ldots ,2L \end{array}$$

where $\lambda =\alpha^{2}\left(L+\kappa \right)-L$, *L* is the dimension of the state vector, and α and κ are the scaling parameters.

**State Prediction:** Propagate sigma points through the nonlinear state transition function

(17) $$\chi_{k|k-1}^{\left(i\right)}=f\left(\chi_{k-1}^{\left(i\right)}\right)$$

Calculate the predicted state mean ${\hat{x}}_{k}^{-}$ and covariance $P_{k}^{-}$

(18) $${\hat{x}}_{k}^{-}=\sum_{i=0}^{2L}W_{m}^{\left(i\right)}\chi_{k|k-1}^{\left(i\right)}$$

(19) $$P_{k}^{-}=\sum_{i=0}^{2L}W_{c}^{\left(i\right)}\left(\chi_{k|k-1}^{\left(i\right)}-{\hat{x}}_{k}^{-}\right){\left(\chi_{k|k-1}^{\left(i\right)}-{\hat{x}}_{k}^{-}\right)}^{⊤}+Q_{k}$$

where $Q_{k}$ is the process noise covariance, $W_{c}^{\left(i\right)}$ and $W_{m}^{\left(i\right)}$ are the weights for the covariance and mean, respectively, defined as

(20) $$\begin{array}{cc} W_{m}^{\left(0\right)} & =\frac{\lambda }{L+\lambda },\;W_{c}^{\left(0\right)}=\frac{\lambda }{L+\lambda }+\left(1-\alpha^{2}+\beta \right) \end{array}$$

(21) $$\begin{array}{cc} W_{m}^{\left(i\right)} & =W_{c}^{\left(i\right)}=\frac{1}{2(L+\lambda )},\;i=1,\ldots ,2L \end{array}$$

Here, β is a constant used to incorporate prior knowledge of the distribution of x (e.g., choosing β=2 is optimal for a Gaussian distribution) [39].

**Measurement Update:** Propagate the sigma points through the nonlinear measurement function

(22) $$\Upsilon_{k}^{\left(i\right)}=h\left(\chi_{k|k-1}^{\left(i\right)}\right)$$

Calculate the predicted measurement mean ${\hat{y}}_{k}$ and its covariance $P_{k}^{y}$

(23) $$\begin{array}{cc} {\hat{y}}_{k} & =\sum_{i=0}^{2L}W_{m}^{\left(i\right)}\Upsilon_{k}^{\left(i\right)} \end{array}$$

(24) $$\begin{array}{cc} P_{k}^{y} & =\sum_{i=0}^{2L}W_{c}^{\left(i\right)}\left(\Upsilon_{k}^{\left(i\right)}-{\hat{y}}_{k}\right){\left(\Upsilon_{k}^{\left(i\right)}-{\hat{y}}_{k}\right)}^{⊤}+R \end{array}$$

where R is the measurement noise covariance.

**Kalman Gain and State Update:** Compute the cross-covariance $P_{xy}$ and the Kalman gain K

(25) $$\begin{array}{cc} P_{xy} & =\sum_{i=0}^{2L}W_{c}^{\left(i\right)}\left(\chi_{k|k-1}^{\left(i\right)}-{\hat{x}}_{k}^{-}\right){\left(\Upsilon_{k}^{\left(i\right)}-{\hat{y}}_{k}\right)}^{⊤} \end{array}$$

(26) $$\begin{array}{cc} K & =P_{xy}{\left(P_{k}^{y}\right)}^{-1} \end{array}$$

Finally, update the state estimate ${\hat{x}}_{k}$ and the covariance $P_{k}$ using the actual measurement $y_{k}$

(27) $$\begin{array}{cc} {\hat{x}}_{k} & ={\hat{x}}_{k}^{-}+K\left(y_{k}-{\hat{y}}_{k}\right) \end{array}$$

(28) $$\begin{array}{cc} P_{k} & =P_{k}^{-}-KP_{k}^{y}K^{⊤} \end{array}$$

These steps collectively define the Unscented Kalman Filter (UKF) algorithm, where stage, from sigma point generation to the final state update, is executed deterministically based on the underlying nonlinear functions. This structured process enables the UKF to deliver recursive state estimates without requiring explicit Jacobians, thereby simplifying implementation while preserving higher-order accuracy.

#### 2.1.3. Cubature Kalman Filter

The Cubature Kalman Filter (CKF) is an advanced variant of the Kalman Filter designed to handle the complexities of nonlinear systems, particularly in high-dimensional spaces. Unlike the Unscented Kalman Filter, which relies on sigma points, the CKF employs a cubature rule based on spherical-radial integration, enhancing its ability to manage system nonlinearities more effectively, thereby improving estimation performance in complex scenarios [40,41]. The following presents a detailed formulation of the CKF, including its prediction and correction phases for a general nonlinear discrete-time system.

For a nonlinear discrete-time system, the CKF operates through the following steps.

**Initialization:** Initialize state and covariance estimates

(29) $$\begin{array}{cc} {\hat{x}}_{0|0} & =E[x_{0}] \end{array}$$

(30) $$\begin{array}{cc} P_{0|0} & =E[\left(x_{0}-{\hat{x}}_{0|0}\right){\left(x_{0}-{\hat{x}}_{0|0}\right)}^{⊤}] \end{array}$$

**Prediction Step:** Compute the cubature points $\chi_{i}$ based on the current state estimate ${\hat{x}}_{k-1|k-1}$ and its covariance $P_{k-1|k-1}$

(31) $$\chi_{i}={\hat{x}}_{k-1|k-1}+\sqrt{P_{k-1|k-1}}\;\zeta_{i}$$

where $\zeta_{i}$ are the cubature points chosen according to the spherical-radial cubature rule [41]. The cubature points are then propagated through the state transition function *f*

(32) $$\chi_{i,k|k-1}=f\left(\chi_{i},u_{k-1}\right)$$

Compute the predicted state mean as

(33) $${\hat{x}}_{k|k-1}=\sum_{i=1}^{2n}\omega_{i}\chi_{i,k|k-1}$$

where $\omega_{i}=\frac{1}{2n}$.

Compute the predicted state covariance as

(34) $$P_{k|k-1}=\sum_{i=1}^{2n}\omega_{i}\left(\chi_{i,k|k-1}-{\hat{x}}_{k|k-1}\right){\left(\chi_{i,k|k-1}-{\hat{x}}_{k|k-1}\right)}^{⊤}+Q_{k-1}$$

**Correction Step:** Propagate the cubature points through the measurement function *h*

(35) $$y_{i,k|k-1}=h\left(\chi_{i}\right)$$

Calculate the predicted measurement mean as

(36) $${\hat{y}}_{k|k-1}=\sum_{i=1}^{2n}\omega_{i}y_{i,k|k-1}$$

Next, compute the innovation covariance $P_{yy,k}$ and cross-covariance $P_{xy,k}$ as

(37) $$\begin{array}{cc} P_{yy,k} & =\sum_{i=1}^{2n}\omega_{i}\left(y_{i,k|k-1}-{\hat{y}}_{k|k-1}\right){\left(y_{i,k|k-1}-{\hat{y}}_{k|k-1}\right)}^{⊤}+R_{k} \end{array}$$

(38) $$\begin{array}{cc} P_{xy,k} & =\sum_{i=1}^{2n}\omega_{i}\left(\chi_{i,k|k-1}-{\hat{x}}_{k|k-1}\right){\left(y_{i,k|k-1}-{\hat{y}}_{k|k-1}\right)}^{⊤} \end{array}$$

Calculate the Kalman gain $K_{k}$ as

(39) $$K_{k}=P_{xy,k}P_{yy,k}^{-1}$$

Finally, the updated state estimate and the state covariance are

(40) $$\begin{array}{cc} {\hat{x}}_{k|k} & ={\hat{x}}_{k|k-1}+K_{k}\left(y_{k}-{\hat{y}}_{k|k-1}\right) \end{array}$$

(41) $$\begin{array}{cc} P_{k|k} & =P_{k|k-1}-K_{k}P_{yy,k}K_{k}^{⊤} \end{array}$$

The CKF algorithm, as detailed above, defines a structured and recursive process for nonlinear state estimation. By leveraging numerical integration through deterministic cubature points, it enables consistent prediction and correction without the need for linear approximations nor heuristic sampling, making it more suitable for a broad range of complex nonlinear system applications.

#### 2.1.4. Ensemble Kalman Filter

The Ensemble Kalman Filter (EnKF), introduced by Evensen in 1994 [42,43], is a Monte Carlo approximation of the traditional Kalman Filter, intended for high-dimensional, nonlinear systems applications [24]. Departing from the standard Kalman filter, which assumes Gaussian distributions to compute single state estimation, the EnKF employs a large ensemble of state estimates to approximate the distribution of possible states. This EnKF solution produced a more robust prediction in complex and uncertain environments [44,45]. The four primary steps in the EnKF process are: initialization, prediction, update, and iteration.

**Initialization:** An ensemble of *N* members $x_{i}^{0}\in R^{n}$ is initialized, where each member represents a possible state of the system.

**Prediction Step:** Propagate each ensemble member $x_{i}^{k}$ using the state dynamics

(42) $$x_{i}^{f,k+1}=f\left(x_{i}^{k}\right)+w_{i}^{k}$$

where *f* represents the system dynamics and $w_{i}^{k}$ the process noise for the *i*th ensemble member.

**Update Step:** Update each predicted ensemble member using the observation

(43) $$x_{i}^{a,k+1}=x_{i}^{f,k+1}+K_{k+1}\left(y_{k+1}-H\left(x_{i}^{f,k+1}\right)+\epsilon_{i}^{k+1}\right)$$

where $x_{i}^{a,k+1}$ is the updated ensemble member, $K_{k+1}$ is the Kalman gain, $y_{k+1}$ is the output, H is the observation operator, and $\epsilon_{i}^{k+1}∼N\left(0,R_{k+1}\right)$ is the measurement noise.

**Kalman Gain Calculation:** Compute the Kalman gain using the forecast covariance matrix $P^{f,k+1}$ derived from the ensemble members

(44) $$K^{k+1}=P^{f,k+1}H^{⊤}{\left(HP^{f,k+1}H^{⊤}+R_{k+1}\right)}^{-1}$$

The forecast covariance matrix is approximated by

(45) $$P^{f,k+1}=\frac{1}{N-1}\sum_{i=1}^{N}\left(x_{i}^{f,k+1}-{\tilde{x}}^{f,k+1}\right){\left(x_{i}^{f,k+1}-{\tilde{x}}^{f,k+1}\right)}^{⊤}$$

where ${\tilde{x}}^{f,k+1}$ is the sample mean of the forecasted ensemble

(46) $${\tilde{x}}^{f,k+1}=\frac{1}{N}\sum_{i=1}^{N}x_{i}^{f,k+1}$$

**Iteration:** The updated ensemble $x_{i}^{a,k+1}$ is then used as the new initial condition for the next iteration of the process.

### 2.2. Joint State and Parameter Estimation Using Kalman Based Algorithms

In joint state and parameter estimation, the system’s state vector is augmented to include both the dynamic states and the unknown parameters to be estimated. The standard filter equations are then applied, modified to facilitate the simultaneous estimation of both quantities, states and parameters, from system measurements.

Let x denote the state vector and θ the model parameters vector; the augmented state vector is then defined as

(47) $$x_{a}=\left[\begin{array}{c} x\\ \theta \end{array}\right]$$

*Note:* When a system includes unknown parameters, even a model that is linear in the state variables may lead to a nonlinear estimation problem once the parameters are treated as additional states. This occurs because the parameters often appear multiplicatively in the system matrices, introducing nonlinear couplings between the original states and the parameters in the augmented model.

By incorporating the dynamics of the parameters into the system and measurement models, the updated representation becomes

(48) $$\begin{array}{cc} x_{a,k+1} & =A_{a}x_{a,k}+B_{a}u_{k}+w_{a}, \end{array}$$

(49) $$\begin{array}{cc} y_{k} & =H_{a}x_{a,k}+v_{k}, \end{array}$$

where $A_{a}$, $B_{a}$, and $H_{a}$ are the augmented state transition, input, and observation matrices, respectively, and $w_{a}$ is the augmented process noise vector. These matrices explicitly combine the nominal system matrices with the parameter-dependent components, embedding the parameter effects directly within the dynamic update. Specifically, $A_{a}$ incorporates both the system dynamics and parameter coupling terms, $B_{a}$ represents the input mapping including parameter dependencies, and $H_{a}$ defines how both the states and parameters influence the measured outputs. This representation preserves the discrete-time state-space structure while enabling simultaneous estimation of both system states and parameters within a unified framework.

Joint state and parameter estimation is widely applied in engineering. For both linear and nonlinear systems, the Joint Extended Kalman Filter (JEKF) is a commonly used method. A typical application is in battery management systems, where the JEKF is used to estimate the state of charge (SOC) and various model parameters [46]. However, in systems with significant nonlinearities, the performance of the JEKF may degrade due to the inaccuracies introduced by the linearization and Jacobian approximation.

To overcome these limitations, the Joint Unscented Kalman Filter (JUKF) is often employed as it avoids linearization by propagating sigma points through the nonlinear system models, capturing the true mean and covariance of the state distribution more accurately [39]. It has been successfully employed in many research studies. For instance, in [47], the JUKF was used for simultaneous state and parameter estimation in vehicle dynamics, enhancing the performance of driver assistance systems under nonlinear conditions. Similarly, in [48], the JUKF was applied to Managed Pressure Drilling (MPD) to estimate downhole states and drilling parameters using topside measurements, demonstrating reliable performance even during transient drilling operations.

An alternative to the JUKF is the Joint Cubature Kalman Filter (JCKF), which replaces sigma points with cubature points based on spherical-radial integration rules [41]. This method retains the benefits of derivative-free estimation while improving numerical stability and accuracy. In [49], the JCKF was employed for estimating vehicle states and road-dependent parameters, such as tire-road friction coefficients, which are critical for adaptive vehicle control under varying road conditions.

The Joint Ensemble Kalman Filter (JEnKF) leverages ensemble-based methods for joint estimation in high-dimensional, nonlinear systems. Rather than relying on analytical approximations of distributions, the JEnKF uses a Monte Carlo ensemble to represent the posterior distribution. In [44], the Joint EnKF (JEnKF) was employed to estimate soil moisture states and land surface parameters, achieving high estimation accuracy. Additional applications of the JEnKF in joint state-parameter estimation can be found in [44,50,51,52].

### 2.3. Dual State and Parameter Estimation

**Figure 1:**
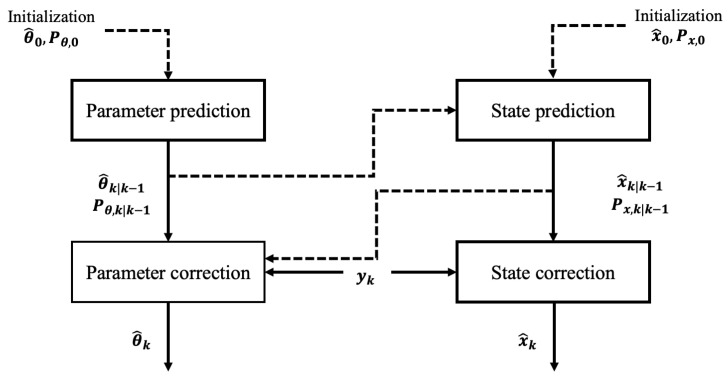
Dual estimation schematic.

In dual estimation, two independent filters operate in parallel: one for state estimation and the other for model parameters identification. The state filter utilizes system measurements and the current model to estimate the dynamic state variables, while the parameter filter relies on the updated state estimates to refine the parameter estimates. This structure enables a decoupled yet interactive estimation process that enhances adaptability and robustness. A schematic of the dual estimation framework is illustrated in Figure 1.

The dual estimation approach has been applied successfully in various engineering domains. For example, in [53], the Dual Extended Kalman Filter (DEKF) was employed to estimate both vehicle states and parameters, specifically tire cornering stiffness, which changes under different driving conditions. By allowing continuous adaptation of the parameters as vehicle dynamics evolved, the method dramatically improved state estimation accuracy and enhanced real-time vehicle control performance.

Similarly, in [54] the Dual Unscented Kalman Filter (DUKF) was utilized for state and parameter estimation in surface-atmosphere exchange processes, addressing missing data in net ecosystem CO_2_ exchange (NEE). The DUKF provided continuous updates that outperformed conventional gap-filling techniques by improving estimation accuracy and reducing subjectivity, while effectively accounting for uncertainties in both model structure and measurements.

In [55], the Dual Cubature Kalman Filter (DCKF) was utilized to estimate the state of charge and circuit model parameters in lithium-ion batteries, delivering accurate and reliable results under varying operational conditions. Additionally, in [56], the Dual Ensemble Kalman Filter (DEnKF) was implemented to enhance streamflow forecasting, enabling simultaneous estimation of hydrological states and parameters, such as soil moisture and storage capacities, within a dynamic model. This approach addressed limitations of traditional calibration techniques, by enabling continuous updates in response to environmental changes.

### 2.4. Comparison

Both joint and dual state-parameter estimation methods aim to recover the same quantities, namely, the system states and the unknown parameters, from noisy measurements. The distinction lies not in the problem formulation but in the computational architecture used to achieve this goal, specifically: (1) In joint estimation, the state and parameter vectors are combined into a single augmented state vector, and one unified filter (e.g., EKF, UKF, CKF, or EnKF) simultaneously estimates them within a single recursive process. (2) In dual estimation, two filters operate in parallel: one estimates the states using the current parameter estimates, while the other updates the parameters based on the most recent state estimates. This decoupling reduces filter coupling errors and often improves numerical stability, at the cost of increased computational complexity and a possible time lag between state and parameter updates.

Numerous research studies have compared the performance of joint and dual estimation approaches across different applications, including chemical processes, vehicle dynamics, and environmental systems [44,49,53,54,56,57,58,59,60]. In [57] the Joint Extended Kalman Filter (JEKF) was evaluated against the Dual Extended Kalman Filter (DEKF) for simultaneous estimation of states and parameters in a highly nonlinear continuous stirred tank reactor (CSTR). The results highlighted the advantages of DEKF, particularly in reducing the risk of filter divergence by decoupling the state and parameter estimation processes. Moreover, the DEKF reduces computational load by allowing the parameter estimator to be turned off once the parameters converge to their optimal values, without compromising the accuracy of state estimation. This feature is particularly beneficial in systems where parameters stabilize over time, thus improving filter efficiency.

Another study in [58] proposed a modified version of the Dual Unscented Kalman Filter (MDUKF), which builds on the DUKF’s ability to deactivate the parameter estimator once sufficient accuracy is achieved. Unlike the standard DUKF, which continuously estimates state and parameter variables separately, the MDUKF improves performance by integrating a refined approach that allows the parameter estimator to be turned off once a satisfactory accuracy level is reached via a selective update mechanism. This strategy significantly reduces computational load while improving estimation performance. The effectiveness of this approach was demonstrated in the dual estimation of vehicle states and critical parameters such as side-slip angle, lateral tire-road forces, and the tire-road friction coefficient.

Similarly, the performance of the JCKF against the DCKF was assessed in [49]. The simulation results indicate that while both methods achieve accurate estimations, the DCKF consistently surpasses the JCKF in terms of accuracy, computational efficiency, and robustness, especially under varying conditions. These findings makes the dual estimation approach the preferred choice for real-time applications, especially in scenarios where environmental conditions are unpredictable.

A concise summary of the main characteristics, advantages, and limitations of the joint and dual Kalman-based estimation methods is provided in Table 2.

### 2.5. Challenges with Kalman-Based Methods

The performance of Kalman-based filters heavily depends on the appropriate selection of the process and measurement noise covariance matrices, Q and R [1]. These matrices define the statistical properties of process and measurement noise, respectively. Achieving the right balance is crucial: if the covariance values are set too low, the filter may become overly sensitive to disturbances, leading to inaccurate estimates. Conversely, excessively high values can result in overly conservative estimates, diminishing the filter’s responsiveness and adaptability [1].

One major challenge is accurately modeling the complex and nonlinear noise sources that are prevalent in real-world applications [61]. Noise characteristics often change over time and are affected by environmental factors and operating conditions, making it challenging to accurately capture their statistical properties. Inaccurate noise modeling can result in incorrect assumptions about system behavior, leading to degraded filter performance or even divergence [62,63].

Another challenge lies in tuning critical algorithm-specific parameters, such as the unscented transform parameters (α, β, and κ) for the Unscented Kalman Filter (UKF) [64,65], the optimal placement and weights of cubature points for the Cubature Kalman Filter (CKF), and the appropriate number of ensemble members for the Ensemble Kalman Filter (EnKF). These parameters are often tuned manually with minimal theoretical guidance, relying on trial and error until satisfactory filter performance is achieved. However, this ad hoc approach is time-consuming and does not guarantee optimal parameter selection, especially given the large number of parameters involved [46].

To address these challenges, several research efforts have proposed systematic approaches [66,67,68]. For example, a reference recursive recipe (RRR) has been developed for tuning Kalman filter covariance matrices, which iteratively updates the matrices based on sample statistics derived from the filter [69,70]. Another approach utilizes particle swarm optimization (PSO) algorithms to fine-tune filter parameters. These algorithms iteratively adjust the covariance matrices at each time step by optimizing a predefined cost function, as demonstrated in [1,71,72]. The approach in [72] utilize a particle swarm optimization (PSO) algorithm to fine-tune Kalman filter parameters, iteratively updating the filter covariance matrices at each time step by minimizing the mean squared error. Additionally, Ref. [64] introduces a systematic framework for tuning the scaling parameters α, β, and κ of the unscented transform in the UKF, providing a structured approach to enhance filter performance while reduce reliance on manual tuning.

These advancements provide promising alternatives to traditional filter parameter selection methods, enabling more efficient and accurate selection and enhancing the robustness of Kalman-based filters in practical applications.

## 3. Simultaneous States and Parameters Estimation: Observer-Based Methods

Observer-based approaches provide an alternative framework to Kalman filtering for the simultaneous estimation of system states and parameters. These methods are grounded in deterministic system theory and are often favored for their conceptual simplicity, reduced computational requirements, and ease of implementation in certain classes of systems. This section presents a range of observer-based techniques that have been explored in the literature for simultaneous state and parameter estimation. For consistency with the formulations in Section 2, all observer-based approaches discussed in this section are derived from the general discrete-time nonlinear system model (3) and (4), with specific assumptions (linearity, time-variation, or parameter dependence) applied as required by each observer type.

### 3.1. Observer-Based Methods for Simultaneous State and Parameters Estimation

State estimation via Luenberger observers was first introduced by D. Luenberger [14]. It typically has the following form

(50) $${\hat{x}}_{k+1}=A{\hat{x}}_{k}+Bu_{k}+L\left(y_{k}-C{\hat{x}}_{k}\right)$$

where ${\hat{x}}_{k}$ is the estimated state, $u_{k}$ is the control input, $y_{k}$ is the system output, A and B are the system matrices, and C is the output matrix. L is the observer gain matrix, selected to place the poles of the error dynamic matrix (A−LC) at a desired location determined by the desired convergence rate of the estimations. One major assumption in the design of the Luenberger observer is that the system model (i.e., A, B, and C matrices) match the true plant dynamics (i.e., Equation (1)).

*Note:* To maintain consistency with the notation used in Section 2, the matrices A, B, and C in Equation (50) correspond respectively to F, B, and H in Equation (1).

The application of Luenberger observers for simultaneous state and parameter estimation in linear time-invariant systems was first explored in the early 1970s [73]. The fundamental approach involved augmenting the observer with integrators to estimate constant parameters alongside system states. Later development enabled the application of these methods to time-varying systems [74].

For linear systems, a simple approach is to augment the state vector with the unknown parameters using an augmented Luenberger observer [75]. An alternative approach involves organizing the unknown parameters into structured uncertainty matrices, which are then identified using a modified Luenberger observer architecture in combination with a parameter estimation algorithm to simultaneously perform state and parameter estimation [15].

For nonlinear systems, adaptive observers [76], high-gain observers [77], and sliding mode observers are popular. These techniques provide robust alternatives for handling system nonlinearities and uncertainties. A common assumption across all these methods is that the parameter vector θ is observable from the available measurements, ensuring the system’s states and parameters can be effectively estimated.

The reviewed literature demonstrates a variety of observer-based approaches for simultaneous state and parameter estimation, offering effective alternatives to stochastic filtering methods, especially in systems where model structure is well-understood and noise characteristics are not dominant.

#### 3.1.1. Augmented Luenberger Observer

Consider the discrete-time linear system defined as

(51) $$\begin{array}{cc} x_{k+1} & =Ax_{k}+Bu_{k}+A_{\theta ,k}\theta_{k}, \end{array}$$

(52) $$\begin{array}{cc} y_{k} & =Cx_{k}, \end{array}$$

where $x_{k}\in R^{n}$ is the state vector, $u_{k}\in R^{m}$ is the control input, $y_{k}\in R^{p}$ is the measurement output, and $\theta_{k}\in R^{q}$ is the vector of unknown parameters. The matrices A, B, and C represent the system dynamics, input, and output, respectively, while $A_{\theta ,k}$ is the parameters matrix, capturing the influence of the parameters on the system dynamics.

In this method, the modified observer equation for the augmented system can be written as [75]

(53) $${\hat{z}}_{k+1}=A\left({\hat{\theta }}_{k}\right){\hat{z}}_{k}+B\left({\hat{\theta }}_{k}\right)u_{k}+L\left(y_{k}-C\left({\hat{\theta }}_{k}\right){\hat{z}}_{k}\right),$$

where ${\hat{z}}_{k}={\left[\begin{array}{cc} {\hat{x}}_{k} & {\hat{\theta }}_{k} \end{array}\right]}^{T}$ is the estimate of the augmented state vector, and L is the observer gain matrix. The gain L is designed to ensure that the matrix $A\left({\hat{\theta }}_{k}\right)-LC\left({\hat{\theta }}_{k}\right)$ is Schur stable. This implies that all the eigenvalues lie strictly inside the unit circle. As in the standard Luenberger observer, the output error term $\left(y_{k}-C\left({\hat{\theta }}_{k}\right){\hat{z}}_{k}\right)$ is used to correct the estimates, driving them towards the true values. The convergence rate of the observer can be adjusted when designing the observer gain L. Common methods to do that is via pole placement or linear matrix inequalities (LMI) [78,79].

The system matrices $A(\theta_{k})$, $B(\theta_{k})$, and $C(\theta_{k})$ of the augmented system in (53) can be written as

(54) $$A\left(\theta_{k}\right)=\left[\begin{array}{cc} A & A_{\theta }\left(\theta_{k}\right)\\ 0 & I \end{array}\right],\;B\left(\theta_{k}\right)=\left[\begin{array}{c} B(\theta_{k})\\ 0 \end{array}\right],\;C\left(\theta_{k}\right)=\left[\begin{array}{cc} C(\theta_{k}) & 0 \end{array}\right].$$

It is commonly assumed that the influence of parameters is incorporated into the state matrix $A(\theta_{K})$. However, in practice, parameters may also affect the input matrix B [15] or the output matrix C [80]. This flexibility allows the observer structure to be adapted to different system representations. However, a notable drawback of this method is its restriction to linear systems, as described in Equations (51) and (52), along with the assumption that parameters are constant or vary slowly ($\dot{\theta }\left(t\right)=0$). When the unknown parameters undergo significant variations, this assumption can degrade performance, preventing the estimates from accurately tracking changes in the true parameters. Moreover, since many real-world systems are inherently nonlinear, directly applying this method often fails to achieve effective state-parameter estimation. In such cases, alternative approaches, such as adaptive observers, are required to overcome these challenges.

#### 3.1.2. Observer-RLSE-CR Method

The Observer-RLSE-CR method integrates deterministic state estimation with adaptive parameter identification in systems with time-varying uncertainties. In this approach, the system matrices are expressed as the sum of a nominal and a perturbation matrix [15]

(55) $$\begin{array}{cc} x_{k+1} & =A\left(k\right)x_{k}+B\left(k\right)u_{k} \end{array}$$

(56) $$\begin{array}{cc} y(k) & =Cx_{k} \end{array}$$

where $x_{k}\in R^{n}$ is the system state, $u_{k}\in R^{m}$ is the system input, and $y_{k}\in R^{p}$ is the measured system output. The system matrices are defined as $A\left(k\right)=A_{0}+\Delta A\left(k\right)\in R^{n\times n}$ and $B\left(k\right)=B_{0}+\Delta B\left(k\right)\in R^{n\times m}$, where $A_{0}$ and $B_{0}$ are the nominal system parameters, and ΔA(k) and ΔB(k) are the unknown time-varying parameters. The system output matrix, $C\in R^{p\times n}$, defines the individual state(s) that are measured.

The study in [15] proposes an Observer-RLSE-CR framework for the joint estimation of system states and parameters. The proposed algorithm integrates a modified Luenberger equation for state estimation, as shown in Equation (57), while concurrently estimating the uncertainty matrices online using recursive least squares estimation with covariance reset (RLSE-CR). Specifically,

(57) $${\hat{x}}_{k+1}=\left(A_{0}+\hat{\Delta A}\left(k\right)\right){\hat{x}}_{k}+L\left(y_{k}-C_{0}{\hat{x}}_{k}\right)+\left(B_{0}+\hat{\Delta B}\left(k\right)\right)u_{k}$$

where ${\hat{x}}_{k}$ is the state estimate, L is the observer gain, $\hat{\Delta A}\left(k\right)$ and $\hat{\Delta B}\left(k\right)$ are the estimates of the system state and input uncertainty matrices, respectively. These time-varying uncertainty estimates are obtained online using the RLSE-CR algorithm, which recursively minimizes a least-squares cost function while implementing a covariance reset mechanism to improve estimator stability and responsiveness. The reset is activated once convergence criteria are met, thereby enhancing adaptability in the presence of parameter drift or abrupt changes.

**Figure 2:**
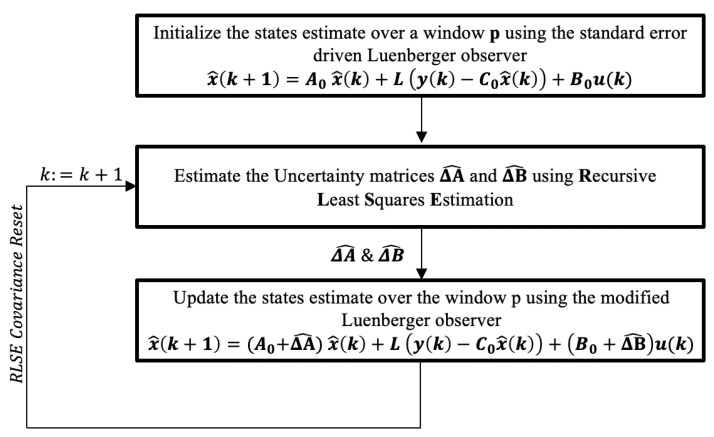
Flowchart of computing the parameter and state estimate using Observer-RLSE-CR Method [15].

The flowchart of the algorithm proposed in [15] is presented in Figure 2. Further insights into the parameter estimation process can be found in [15]. Additionally, a systematic method is introduced for selecting the sliding window size *p* in the RLSE-CR procedure, based on the desired convergence rate and system settling time as well.

A comparison between the Observer-RLSE-CR estimator, the augmented Kalman filter, and the Kalman filter coupled with RLSE is provided in [15]. While all the algorithms effectively estimate both states and parameters, the Observer-RLSE-CR has shown benefits over the Kalman-based filters in capturing uncertainties within the input uncertainty matrix ΔB.

This approach presents several advantages over the augmented Luenberger observer. It not only enables accurate joint estimation of system states and time-varying parameters but also effectively mitigates noise introduced by measurement sensors. However, a key limitation of this method is its restriction to linear systems.

#### 3.1.3. High Gain Observer

High-gain observers are widely used for nonlinear systems or systems with varying dynamics [81,82]. It employs a large observer gain to amplify the output error, enabling rapid corrections of the estimates and facilitating fast convergence to the true values. In linear systems, a standard augmented Luenberger observer formulation is generally adopted, with the observer gain set to a high level. For nonlinear systems, a more structured approach is required. This often involves transforming the system into an observable canonical form and defining an appropriate parameter estimation law before applying the high-gain observer.

Consider the discrete-time nonlinear system defined by

(58) $$\begin{array}{cc} x_{k+1} & =f(x_{k},u_{k},\theta_{k}) \end{array}$$

(59) $$\begin{array}{cc} y_{k} & =h(x_{k}) \end{array}$$

where $x_{k}\in R^{n}$ is the system state, $u_{k}\in R^{m}$ is the system input, $y_{k}\in R^{p}$ is the system output, $\theta_{k}\in R^{q}$ is the unknown parameters vector, and *f* and *h* are nonlinear functions describing the system dynamics and output behavior. The parameters may be constant or slowly varying such that $\theta_{k}=g\left(\theta_{k}\right)$, where *g* is the dynamic law governing the parameters variation over time.

When dealing with constant parameters, i.e., $\theta_{k+1}=\theta_{k}$, the estimation process is simplified. An immediate method for the concurrent system state and unknown parameters estimation is to augment the state vector with the unknown parameters [77]. Defining the augmented state vector as

(60) $$z_{k}=\left[\begin{array}{c} x_{k}\\ \theta_{k} \end{array}\right]\in R^{p+q}$$

Thus, the high-gain observer for the nonlinear augmented system can be written as

(61) $${\hat{z}}_{k+1}=\left[\begin{array}{c} f({\hat{x}}_{k},u_{k},{\hat{\theta }}_{k})\\ {\hat{\theta }}_{k} \end{array}\right]+L\left(y_{k}-h\left({\hat{x}}_{k}\right)\right)$$

where L is the high-gain observer matrix for the augmented system. The innovation term $\left(y_{k}-h\left({\hat{x}}_{k}\right)\right)$ corrects the estimates based on output discrepancies.

For time-varying parameters, a separate adaptive update law is introduced to allow the observer to track parameter changes, as follows

(62) $${\hat{\theta }}_{k+1}={\hat{\theta }}_{k}+\gamma_{k}\left(y_{k}-h\left({\hat{x}}_{k}\right)\right)$$

Here, $\gamma_{k}$ is a time-varying learning rate that controls the adaptation speed. The innovation term continues to serve as the correction signal, enabling the parameter estimate to follow dynamic changes effectively.

Meanwhile, the state estimates continue to evolve based on the standard high-gain observer formulation, using the estimated parameters to generate forward predictions, as follows

(63) $${\hat{x}}_{k+1}=f\left({\hat{x}}_{k},u_{k},{\hat{\theta }}_{k}\right)+L_{x}\left(y_{k}-h\left({\hat{x}}_{k}\right)\right)$$

where $L_{x}$ is the high-gain matrix for the state estimation.

Another approach that can be adopted for the joint state-parameter estimation is concurrently employing a high-gain observer (i.e., Equation (63)) for state estimation and another estimator such as a Kalman filter or a Recursive Least Squares for parameter estimation.

One drawback of high-gain observers is their heightened sensitivity to modeling errors and measurement noise. The large gains can amplify noise, leading to reduced estimation accuracy and diminished performance. Additionally, high-gain observers often require the system to be expressed in a particular form, such as an observable canonical form or strict-feedback form, thereby limiting their applicability to certain systems.

#### 3.1.4. Sliding Mode Observer

State estimation via sliding mode observer (SMO) was first introduced in the mid 1980s [83,84]. For the system $x_{k+1}=Ax_{k}+Bu_{k}$, $y_{k}=Cx_{k}$, the SMO has a similar form to the standard observer with a replacement of the innovation term with a discontinuous switching function as shown below [84]

(64) $${\hat{x}}_{k+1}=A{\hat{x}}_{k}+Bu_{k}+L\;\mathrm{sign}\left(y_{k}-C{\hat{x}}_{k}\right)$$

The sliding mode term $L\;\mathrm{sign}(y_{k}-C{\hat{x}}_{k})$ enhances the observer’s capacity to manage uncertainties and disturbances by introducing a sliding surface that forces the estimation error to converge to zero in finite time. However, in practical implementations, this mechanism can lead to undesirable chattering effects. To alleviate this, practitioners often replace the switching term with a saturation function, thereby reducing chattering while preserving robustness. Thus, the modified SMO can be written in the form

(65) $${\hat{x}}_{k+1}=A{\hat{x}}_{k}+Bu_{k}+L\;\mathrm{sat}\left(e_{k},\epsilon \right)$$

where $e_{k}=y_{k}-C{\hat{x}}_{k}$ is the estimation error and ϵ is a small positive threshold used to reduce the effect of chattering. The saturation function is defined as [85,86]

(66) $$\mathrm{sat}\left(e_{k},\epsilon \right)=\left\{\begin{array}{cc} \frac{e_{k}}{\epsilon }, & \mathrm{if}\;|e_{k}|<\epsilon \\ \mathrm{sign}(e_{k}), & \mathrm{otherwise} \end{array}\right.$$

Various strategies have been proposed to extend SMOs for joint estimation of states and parameters. One approach involves augmenting the state vector with unknown parameters, treating them as additional state variables to be estimated using a single SMO framework [87]. Another method employs dual SMO structures, with one observer dedicated to state estimation and the other to parameter identification, operating in parallel [88]. An alternative strategy uses an adaptive SMO for state estimation coupled with a separate online parameter estimator. In this configuration, the parameter estimator continuously updates the model parameters in real time, and the observer adapts accordingly [89].

#### 3.1.5. Adaptive Observer

Adaptive observer is commonly used when dealing with nonlinear time-varying systems [74,90,91]. Consider the nonlinear system defined in Equations (58) and (59). The observer for state estimates can be represented with the following equations

(67) $${\hat{x}}_{k+1}=f\left({\hat{x}}_{k},u_{k},{\hat{\theta }}_{k}\right)+L_{k}e_{k}$$

where $L_{k}$ is the nonlinear, time-varying observer gain, and $e_{k}=y_{k}-{\hat{y}}_{k}$ is the estimation error. For adaptive observers, an adaptation law is designed to update the parameter estimates as follows

(68) $${\hat{\theta }}_{k+1}={\hat{\theta }}_{k}+\Gamma_{k}\Phi_{k}e_{k}$$

where $\Gamma_{k}$ is a positive definite matrix controlling the rate of parameter adaptation and $\Phi_{k}$ is the regressor related to the system dynamics. For simple problems, $\Gamma_{k}$ is often set constant such that

$$\Gamma =\left[\begin{array}{cccc} \gamma_{1} & 0 & \ldots & 0\\ 0 & \gamma_{2} & \ddots & 0\\ \vdots & \ddots & \ddots & \vdots \\ 0 & 0 & \ldots & \gamma_{q} \end{array}\right]$$

where $\gamma_{i}>0$ are constants that determine the adaptation speed of each parameter $\theta_{i}$. Larger values of $\gamma_{i}$ lead to faster convergence but may also amplify noise and destabilize the system if set too high.

In more advanced implementations, time-varying gain matrices $\Gamma_{k}$ are adopted. One strategy involves decreasing the values of $\Gamma_{k}$ as the estimation error diminishes, helping to stabilize the adaptation over time. Another common method leverages Lyapunov stability theory, for a guaranteed asymptotic stability of the estimation error [90,92].

Defining the Lyapunov function

(69) $$V_{k}=e_{k}^{T}Pe_{k}+{\tilde{\theta }}_{k}^{T}\Gamma_{k}^{-1}{\tilde{\theta }}_{k}$$

where P is a symmetric positive definite matrix, and ${\tilde{\theta }}_{k}=\theta -{\hat{\theta }}_{k}$ is the parameter estimation error.

Stability is ensured by choosing $\Gamma_{k}$ such that the Lyapunov difference satisfies: $\Delta V_{k}=V_{k+1}-V_{k}<0$ [93].

Adaptive observers are particularly effective in nonlinear, time-varying settings to achieve accurate state and parameter estimation. They rely on an adaptive law that continuously updates parameters over time. The key features of their design include the time-varying observer gain $L_{k}$, the adaptation law driven by the estimation error $e_{k}$, and the parameter update rate $\Gamma_{k}$, which may be constant or time-varying, adaptively tuned to maintain stability and desired performance.

### 3.2. Challenges with Observer-Based Methods

The performance of the Luenberger observer depends heavily on the accuracy of the system model. Modeling uncertainties or incorrect system dynamics can significantly degrade its estimation capabilities, and in some cases, lead to divergence [94]. Furthermore, a major limitation of observer-based methods is their sensitivity to noise, as they do not explicitly incorporate process and measurement noise the way Kalman-based filters do. This makes them particularly vulnerable in noisy environments, often resulting in biased or noisy estimates of states and parameters.

High-gain observers are especially sensitive to measurement noise. Although the use of large gains accelerates convergence, it also amplifies noise, resulting in a trade-off between convergence speed and robustness. Proper tuning of the observer gain is therefore critical, as excessive gain values may destabilize the estimation process or introduce significant noise artifacts into the state estimates.

Sliding mode observers face their own set of challenges, notably the chattering effect caused by high-frequency switching nature of the sliding mode correction term. This can introduce undesirable oscillations that can be detrimental to physical systems. Furthermore, designing effective SMOs often requires careful selection of the sliding surface and observer parameters, typically achieved through trial-and-error procedures, which further complicates practical implementation and tuning.

Adaptive observers pose unique challenges as well. One key requirement is the presence of persistent excitation in the system inputs to ensure convergence of parameter estimates. If this condition is not met, the observer may fail to adequately capture parameter dynamics, resulting in poor estimation accuracy. Additionally, the design of the adaptation gain matrix must balance speed of convergence with robustness to noise and modeling uncertainties.

Finally, across all observer types, another common limitation is their dependence on accurate initial conditions. Poorly initialized state or parameter estimates can lead to slow convergence, large transient errors, or in some cases, complete divergence of the estimation process.

## 4. Comparative Discussion of Kalman-Based and Observer-Based Approaches

From a broader perspective, Kalman-based and observer-based approaches represent two complementary philosophies in simultaneous state and parameter estimation. Kalman-based filters, rooted in stochastic estimation theory, explicitly account for process and measurement noise through covariance modeling and probabilistic estimation. When the statistical properties of the noise are well characterized, these filters provides statistically optimal estimates in the minimum mean-square error sense. The probabilistic foundation enables Kalman-based algorithms to achieve high robustness against random disturbances, measurement uncertainties, and modeling imperfections. However, these advantages come at the expense of increased computational cost and sensitivity to incorrect noise covariance tuning or modeling errors, which can lead to divergence or degraded performance, particularly in highly nonlinear or time-varying systems.

Observer-based methods, by contrast, are grounded in deterministic system theory. They reconstruct system states and parameters through feedback mechanisms that exploit the structure of the underlying model rather than probabilistic assumptions. Because they do not rely on explicit noise statistics, observers are often applied in systems where the model dynamics are well characterized but noise properties are uncertain or difficult to quantify. While this independence from statistical noise modeling simplifies implementation, it also limits robustness to measurement disturbances. Nevertheless, deterministic observers generally exhibit faster convergence, simpler implementation, and lower computational requirements than their Kalman-based counterparts. However, they lack built-in uncertainty quantification mechanisms (e.g., they do not, by construction, produce uncertainty measures such as covariance matrices or confidence intervals) and their performance may degrade in the presence of significant measurement noise, unmodeled dynamics, or parameter drift.

In practical terms, the selection between Kalman-based and observer-based approaches depends on the nature of the system and the information available about its uncertainty sources. For systems dominated by stochastic disturbances, where statistical modeling is feasible and computational resources are sufficient, Kalman-based filters remain the preferred choice. Conversely, for systems with reliable deterministic models, limited sensor data, or stringent real-time constraints, observer-based approaches often provide a more efficient and flexible solution. A concise comparison of the key characteristics, advantages, and limitations of Kalman-based and observer-based approaches is presented in Table 3.

Recent studies have also explored hybrid estimation frameworks that integrate the statistical optimality of Kalman filtering with the structural adaptability of deterministic observers. Such hybrid schemes have been proposed across various domains, including vehicle dynamics, robotics, and energy systems, to enhance robustness and estimation accuracy under mixed stochastic and deterministic uncertainties [88,95,96,97]. These hybrid architectures show promising directions for achieving improved estimation accuracy and stability, particularly in nonlinear systems with time-varying parameters.

## 5. Conclusions

Presented in this manuscript is a comprehensive review and a comparative analysis of various methodologies explored in the literature for the simultaneous estimation of system states and parameters, focusing on approaches based on Kalman filters and Luenberger observers. While Kalman-based methods excel in handling stochastic noise, observer-based methods offer simplicity and efficiency in deterministic settings.

The methods reviewed are generally categorized into three main approaches:

- **Augmented State Approach:** The unknown parameters are treated as additional states within the system, leading to an augmented state vector. An observer is then designed to estimate the full set of states, including both the system states and the unknown parameters. This method is advantageous in systems where the states and parameters are interdependent, but the complexity of the augmented observer increases with the number of parameters, which can affect computational efficiency and robustness to noise.
- **Decoupled Estimation Approach:** The estimation tasks for the unknown parameters and the system states are decoupled, and two separate observers are run concurrently, one for state estimation and the other for parameter estimation. This approach simplifies the observer design by separating the two estimation problems. However, challenges arise when strong interactions exist between the system states and parameters, which can lead to inaccuracies or slow convergence.
- **Parameter Identification Coupled with State Estimation:** A parameter identification technique is used to estimate the unknown parameters, which is then coupled with a state observer for state estimation. This approach leverages well-established parameter estimation techniques, such as least squares, in conjunction with traditional state observers. While this method can be efficient, its accuracy and convergence are highly dependent on the parameter identification process and the model’s sensitivity to parameter changes.

The challenges and limitations associated with each estimation method have been explored. A significant challenge is noise sensitivity, particularly in methods like the Luenberger observer, which rely heavily on accurate model assumptions. Robustness to noise is a critical factor, especially in real-world systems where measurements are often noisy or uncertain. Additionally, the computational complexity of the methods varies significantly, with some requiring substantial resources due to the complexity of the observer design or the number of parameters involved.

Each method offers distinct advantages and trade-offs depending on the system’s characteristics and performance requirements. For example, the augmented state approach provides a unified framework for simultaneous state and parameter estimation but may impose a higher computational burden. On the other hand, decoupled approaches can reduce computational effort but may struggle when state and parameter dynamics are strongly coupled.

The selection of the most appropriate method ultimately depends on several factors, including the quality and quantity of available data, the complexity of the system dynamics, the accuracy of the system model, and the desired level of computational efficiency. Future research could focus on hybrid approaches that integrate the strengths of multiple methods, as well as adaptive techniques that dynamically adjust the estimation strategy based on real-time data characteristics and evolving system behavior. These advancements could further enhance the robustness and applicability of state and parameter estimation methods in complex, noisy environments.

### Future Research Directions

Although significant progress has been made in simultaneous state and parameter estimation using Kalman filters and observer-based approaches, several important research challenges remain open. One promising direction is the development of hybrid stochastic-deterministic frameworks that leverage the complementary strengths of both paradigms. For example, embedding observer feedback structures within Kalman-based filters could improve numerical stability and convergence under parameter uncertainty, while integrating covariance adaptation into deterministic observers may enhance robustness to noise and unmodeled dynamics.

A second key challenge concerns the treatment of strongly coupled states and parameters (e.g, cases when state and parameters contributions to measured outputs are difficult to distinguish), where interdependence reduces observability and can lead to estimator divergence or slow convergence. Future research should focus on systematic observability analysis, parameter sensitivity quantification, and coupling-decoupling strategies that preserve estimation accuracy while maintaining computational tractability.

Another important direction involves handling fast time-varying parameters. Most existing simultaneous state and parameter estimation algorithms assume slowly varying or piecewise-constant (quasi-static) parameters (i.e., $\theta_{k+1}=\theta_{k}$ within the estimation window), which limits their applicability in systems with rapid parameter evolution. Addressing this issue requires adaptive observers and Kalman filter variants capable of tracking fast parameter dynamics without compromising numerical stability or state accuracy.

Furthermore, data-driven and learning-assisted estimation techniques are expected to play an increasingly important role. The fusion of physics-based observers with neural or regression-based parameter estimators could enable real-time adaptation to nonlinear or partially known dynamics while maintaining interpretability and physical consistency.

From a computational perspective, the scalability of simultaneous state and parameter estimation algorithms to high-dimensional systems remains an unsolved challenge. Efficient reduced-order, distributed implementations, particularly for systems with large augmented state vectors, represent a critical area for future exploration.

## Figures and Tables

**Table 1:** Kalman filter algorithm.

Prediction:	
Predicted state estimate	${\hat{x}}_{k}^{-}=F_{k-1}{\hat{x}}_{k-1}^{+}+B_{k-1}u_{k-1}$
Predicted error covariance	$P_{k}^{-}=F_{k-1}P_{k-1}^{+}F_{k-1}^{⊤}+Q_{k-1}$
**Update:**	
Measurement residual	${\tilde{y}}_{k}=y_{k}-H_{k}{\hat{x}}_{k}^{-}$
Kalman gain	$K_{k}=P_{k}^{-}H_{k}^{⊤}{\left(H_{k}P_{k}^{-}H_{k}^{⊤}+R_{k}\right)}^{-1}$
Updated state estimate	${\hat{x}}_{k}^{+}={\hat{x}}_{k}^{-}+K_{k}{\tilde{y}}_{k}$
Updated error covariance	$P_{k}^{+}=\left(I-K_{k}H_{k}\right)P_{k}^{-}$

**Table 2:** Comparison of joint and dual Kalman-based estimation methods.

Filter Type	Scheme	Computation	Advantages	Limitations
**EKF**	**Joint (JEKF)**	Low-Medium	Compact implementation.	Linearization errors; loss of accuracy for strong nonlinearities.
	**Dual (DEKF)**	Medium	Improved stability via state-parameter decoupling.	Higher cost; potential delay between estimates.
**UKF**	**Joint (JUKF)**	Medium	Jacobian-free; second-order accuracy for smooth dynamics.	Sensitive to scaling parameters (α,β,κ).
	**Dual (DUKF)**	Med-High	Handles strong nonlinear coupling robustly.	Requires two filters; increased tuning and cost.
**CKF**	**Joint (JCKF)**	Med-High	Numerically stable; suitable for higher dimensions.	Cubature integration increases computation.
	**Dual (DCKF)**	High	Strong noise attenuation; robust under parameter drift.	Sensitive to covariance tuning; computationally heavy.
**EnKF**	**Joint (JEnKF)**	High	Scalable to large stochastic systems.	Sampling noise; large ensemble needed.
	**Dual (DEnKF)**	High-Very High	Robust to model uncertainty; adaptive to time-varying parameters.	High computational load; possible ensemble divergence.

**Table 3:** Comparison of Kalman-based and Observer-based approaches for simultaneous state and parameter estimation.

Aspect	Kalman-Based Methods	Observer-Based Methods
**Theoretical Basis**	Stochastic estimation theory (probabilistic, covariance-driven).	Deterministic system theory (model-structure-driven).
**Noise Handling**	Explicitly models process and measurement noise via covariance matrices (Q, R).	Implicitly accounts for disturbances through feedback; no explicit noise statistics.
**Optimality**	Statistically optimal or near-optimal under Gaussian noise assumptions.	Not statistically optimal; relies on deterministic stability and observability conditions.
**Computational Complexity**	Moderate to high (matrix operations, covariance updates, or ensemble propagation).	Generally low, via simpler recursive structure.
**Model Dependence**	Robust to moderate model mismatch if noise statistics are accurate.	Sensitive to modeling errors; accuracy depends on precise system representation.
**Uncertainty Quantification**	Covariance-based uncertainty metrics.	Does not directly quantify estimation uncertainty.
**Nonlinearity Handling**	Extended, Unscented, Cubature, and Ensemble variants manage nonlinearities effectively.	Nonlinear extensions via adaptive, high-gain, or sliding-mode observers.
**Tuning Parameters**	Requires careful selection of Q, R, and algorithm-specific parameters.	Primarily tuned via gain matrices or adaptation laws.
**Typical Applications**	Navigation, tracking, robotics, battery management, hydrology.	Vehicle dynamics, process control, electromechanical systems, robotics.

## Data Availability

No new data were created or analyzed in this study. Data sharing is not applicable to this article.

## Abbreviations

The following abbreviations are used in this manuscript:

KF	Kalman Filter
EKF	Extended Kalman Filter
UKF	Unscented Kalman Filter
CKF	Cubature Kalman Filter
EnKF	Ensemble Kalman Filter
JEKF	Joint Extended Kalman Filter
JUKF	Joint Unscented Kalman Filter
JCKF	Joint Cubature Kalman Filter
JEnKF	Joint Ensemble Kalman Filter
DEKF	Dual Extended Kalman Filter
DUKF	Dual Unscented Kalman Filter
DCKF	Dual Cubature Kalman Filter
DEnKF	Dual Ensemble Kalman Filter
LSE	Least Squares Estimation

RLSE	Recursive Least Squares Estimation
RLSE-CR	Recursive Least Squares Estimation with Covariance Reset
SMO	Sliding Mode Observer
PSO	Particle Swarm Optimization
RRR	Reference Recursive Recipe
LMI	Linear Matrix Inequality
PID	Proportional-Integral-Derivative controller
